# Role of myocardial microRNAs in the long-term ventricular remodelling of patients with aortic stenosis

**DOI:** 10.1093/ehjopen/oeae060

**Published:** 2024-07-24

**Authors:** André F Gabriel, Marina C Costa, Daniel Caldeira, Rui Plácido, Joana Rigueira, Pedro Carrilho-Ferreira, Susana Gonçalves, Ricardo Ferreira, Ângelo Nobre, Fausto J Pinto, Francisco J Enguita, Ana G Almeida

**Affiliations:** Instituto de Medicina Molecular João Lobo Antunes, Faculty of Medicine, Lisbon University, Av. Prof. Egas Moniz, 1649-028 Lisbon, Portugal; Instituto de Medicina Molecular João Lobo Antunes, Faculty of Medicine, Lisbon University, Av. Prof. Egas Moniz, 1649-028 Lisbon, Portugal; Heart and Vessels Department, University Hospital ULS Santa Maria, CCUL@RISE, Faculty of Medicine, Lisbon University, Av. Prof. Egas Moniz, 1649-028 Lisbon, Portugal; Heart and Vessels Department, University Hospital ULS Santa Maria, CCUL@RISE, Faculty of Medicine, Lisbon University, Av. Prof. Egas Moniz, 1649-028 Lisbon, Portugal; Heart and Vessels Department, University Hospital ULS Santa Maria, CCUL@RISE, Faculty of Medicine, Lisbon University, Av. Prof. Egas Moniz, 1649-028 Lisbon, Portugal; Heart and Vessels Department, University Hospital ULS Santa Maria, CCUL@RISE, Faculty of Medicine, Lisbon University, Av. Prof. Egas Moniz, 1649-028 Lisbon, Portugal; Heart and Vessels Department, University Hospital ULS Santa Maria, CCUL@RISE, Faculty of Medicine, Lisbon University, Av. Prof. Egas Moniz, 1649-028 Lisbon, Portugal; Heart and Vessels Department, University Hospital ULS Santa Maria, CCUL@RISE, Faculty of Medicine, Lisbon University, Av. Prof. Egas Moniz, 1649-028 Lisbon, Portugal; Heart and Vessels Department, University Hospital ULS Santa Maria, CCUL@RISE, Faculty of Medicine, Lisbon University, Av. Prof. Egas Moniz, 1649-028 Lisbon, Portugal; Heart and Vessels Department, University Hospital ULS Santa Maria, CCUL@RISE, Faculty of Medicine, Lisbon University, Av. Prof. Egas Moniz, 1649-028 Lisbon, Portugal; Instituto de Medicina Molecular João Lobo Antunes, Faculty of Medicine, Lisbon University, Av. Prof. Egas Moniz, 1649-028 Lisbon, Portugal; Heart and Vessels Department, University Hospital ULS Santa Maria, CCUL@RISE, Faculty of Medicine, Lisbon University, Av. Prof. Egas Moniz, 1649-028 Lisbon, Portugal

**Keywords:** Aortic stenosis, Cardiac remodelling, MicroRNA, Prognosis

## Abstract

**Aims:**

We hypothesize that miRs are key players in the dynamics of the hypertrophy phenotype in aortic stenosis (AS) patients. In our study, we aimed to identify the transcriptional patterns (protein-coding transcripts and miRs) from myocardial sample biopsies that could be associated with the absence of left ventricular (LV) mass regression after aortic valve replacement (AVR) in patients with severe AS and LV hypertrophy.

**Methods and results:**

We prospectively included 40 patients with severe AS, LV hypertrophy, and preserved ejection fraction undergoing AVR. Myocardial biopsies obtained during surgery were analysed for transcriptomic analysis performed by next-generation sequencing. At a 1-year follow-up, no hypertrophy reversal was observed in about half of the patients in the absence of patient–prosthesis mismatch and prosthesis dysfunction of uncontrolled hypertension. Predictors of mass regression were assessed from clinical, echocardiographic, and biochemical variables as well as from 300 miRs obtained from myocardial specimens, allowing the identification 29 differentially expressed. miR-4709-3p was found as a positive independent predictor of hypertrophy regression together with high-sensitivity troponin T (cTNT-hs) as a negative predictor. Gene transcripts RFX1, SIX5, MAPK8IF3, and PKD1 were predicted as simultaneous targets of five upregulated miRs suggesting its importance in LV hypertrophy.

**Conclusion:**

In our cohort, tissue miR-4709-3p and cTNT-hs were independent predictors of hypertrophy regression. The hypertrophy reversal process will likely depend from a complex network where miRNAs may have an important role, allowing a potential opportunity for therapy.

## Graphical Abstract

**Graphical Abstract:**
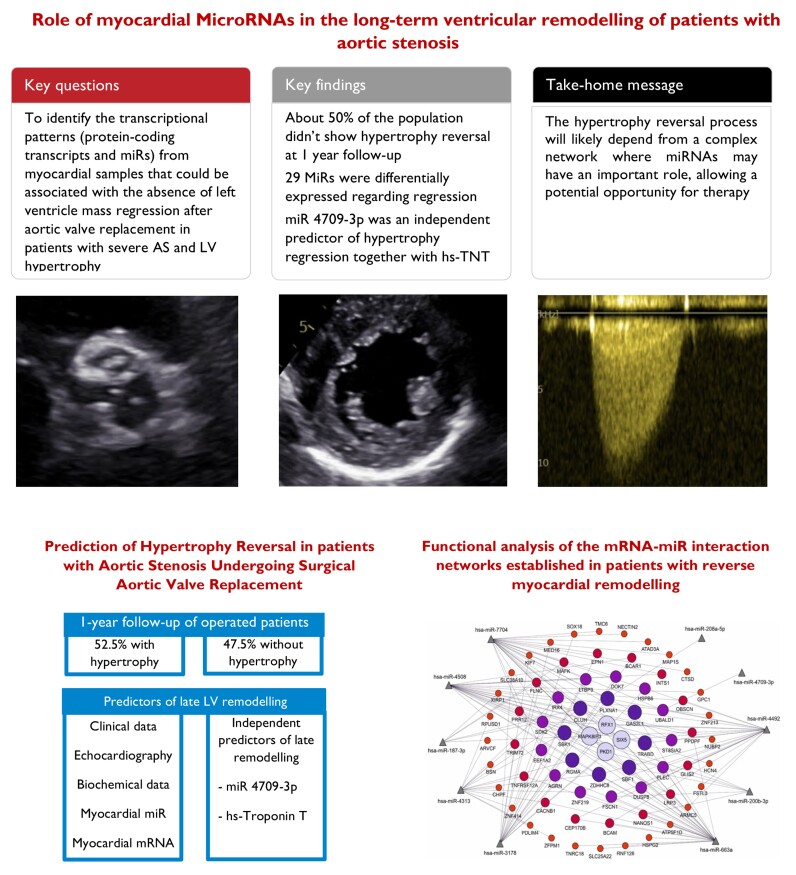

## Introduction

Aortic stenosis (AS) is a progressive degenerative heart disease with a high prevalence in western countries, characterized by left ventricular (LV) systolic obstruction. When severe and symptomatic, mortality is high and the only effective therapy for improving survival is aortic valve replacement (AVR).^1,2^ As a pressure overload disease, AS typically generates a hypertrophic response, believed to be compensatory and beneficial, but that has been shown to be pathological. This response elicits an upregulation of foetal genes^3^ and induces fibrosis, cardiac remodelling, apoptosis, and microvascular ischaemia, leading eventually to ventricular dysfunction, heart failure, and death.^4–6^

In patients with severe AS, there is marked variation regarding the magnitude of the hypertrophic response, suggesting a possible genetic regulatory mechanism.^7–9^ Hypertrophy is absent in 10–20% of patients, who have shown a more benign prognosis than the ones with hypertrophy.^9^

Persistence of LV hypertrophy after AVR has been identified as one of the main limiting factors for the long-term outcome of AS patients, predicting increased morbidity and mortality.^10,11^ On the other hand, recent studies have shown reduced hospitalizations and increased survival in patients showing reverse remodelling, while the maximum LV mass regression occurs within the first 6 months to 1 year after aortic valve surgery.^12,13^ Advanced age, obesity, sex, hypertension, and increased arterial stiffness have been accepted as associated contributors to the pattern of the hypertrophic response and the absence of reverse remodelling after AVR, but the underlying mechanisms are largely unknown.^14^

MicroRNAs (miRs) are small non-coding RNAs generated from specific transcriptional units in eukaryotic genomes that act as negative post-transcriptional regulators of the expression of coding transcripts.^15^ The involvement of miRs in the development and pathologies related to the cardiovascular system has been extensively documented.^16,17^ In the context of AS, miRs play crucial roles in various stages of the disease, influencing its development, progression, and establishment.^18^ Conversely, downregulated miR-30a aggravates pressure overload-induced cardiomyocyte hypertrophy by activating autophagy.^18^ As AS progresses, and the heart compensates for the narrowed valve, miR-21^19^ and miR-34a^20^ are described to promote collagen deposition within the myocardium, contributing to the myocardial stiffening and impairing contractile function, a hallmark of advanced AS. In response to the pressure overload imposed by AS, the ventricle undergoes structural and functional adaptations.

Several studies, describing the potential association of specific miRs with LV hypertrophy and post-AVR remodelling in AS, have been performed both in animal models and human patients.^21–24^ However, results have been conflicting because studies were uncontrolled, and no pre-test was performed for selecting the candidates’ miRs regarding the post-AVR reverse remodelling.^22,25^

We hypothesize that miRs are key players in the dynamics of the hypertrophy phenotype in AS patients. The identification of specific miRs and related transcripts involved in the non-regression patterns could open the opportunity to identify therapies acting on the involved miRs in order to antagonize LV hypertrophy with benefits on prognosis or, otherwise, propose earlier AVR.

Following our hypothesis, we aimed performing a complete transcriptomic analysis in myocardial sample biopsies from patients with severe AS that were referred for AVR, with the objective of identifying the transcriptional pattern that could be associated with the LV hypertrophy development and the absence of mass regression after AVR. This transcriptomic study should cover both mRNA transcripts and miRs for extracting potential functional relationships between both groups.

## Methods

### Study population

We prospectively included 72 consecutive patients with severe AS and preserved ejection fraction (EF) undergoing AVR surgery, according to current indications at a tertiary university hospital. Patients with more than mild aortic or mitral regurgitation, significant coronary artery disease, previous myocardial infarction or coronary intervention, cardiomyopathies, oncologic diseases, renal or hepatic dysfunction, and atrial fibrillation were excluded from the study. Patients with the absence of LV hypertrophy before surgery and with early post-operative criteria for patient–prosthesis mismatch were also excluded.

### Study design

All included patients underwent a pre-operative evaluation with clinical data, echocardiographic study, and biochemical assessment including natriuretic peptides. During surgery for valvular replacement, a myocardial specimen was obtained. Patients were evaluated at 1-year follow-up for clinical data and echocardiography.

### Clinical variables

Demographics (sex, age), body surface area (BSA), history of hypertension, and other risk factors (dyslipidaemia, diabetes, and smoking habits) were registered. Blood pressure was measured at baseline.

### Biochemical data

At baseline, the following variables were obtained: haemoglobin, glomerular filtration rate (GFR), fasting glycaemia, high-sensitivity troponin T (cTNT-hs), and NT-proBNP.

### Echocardiography

Following the diagnosis of severe AS, according to current guidelines^26^ [aortic valve area (AVA) <1 cm^2^ or 0.6 cm^2^/m^2^, mean transvalvular aortic velocity >40 mmHg] with preserved EF, pre-operative and late follow-up echocardiograms were obtained for the assessment of LV end-diastolic (LVEDV) and end-systolic volumes, EF, and LV mass using the Devereux formula. Left ventricle end-diastolic volume and mass were indexed to BSA (iLVEDV and iMass, respectively). The threshold for increased mass was defined according to indexed values and sex following current guidelines, as >95 g/m^2^ for women and >115 g/m^2^ for men.^27^ Other variables obtained at baseline were as follows: AVA and indexed AVA (iAVA), left atrial volume (LAV and iLAV, as indexed to BSA), LV global longitudinal strain (GLS), myocardial global work efficiency (GWE),^28^ stroke volume (SV), and indexed stroke value (iSV). In the late follow-up, LV mass remodelling was analysed using the following criteria: absence of regression to normal using normal reference of indexed values to BSA according to sex and regression of <14% from indexed mass values.^29^ Imaging acquisition parameters were applied according to the European Association of Cardiovascular imaging recommendations. All measurements were performed by two independent echocardiographers with >10 years of practice. A General Electric Vivid 7 equipment was used for image acquisition, and an EchoPAC workstation was used for data analysis (GE Healthcare, Milwaukee, USA).

### Myocardial tissue sampling

In all patients, myocardial biopsies were taken during surgery from the septal LV wall and the samples were immediately frozen upon collection in an RLT buffer (QIAGEN) containing 10% β-mercaptoethanol and stored at −80°C until their analysis.

### Next-generation sequencing

Transcriptomic analysis was performed by next-generation sequencing by the Illumina platform using a paired-end strategy and starting from total RNA preparations that were extracted and purified from cardiac biopsies using a Qiagen RNAeasy Mini Kit. RNA quality was checked by capillary electrophoresis, ensuring a RNA integrety number higher than 7.5. Pair-ended sequencing libraries were prepared and analysed by standard protocols using the manufacturer recommendations, ensuring an average output of 60 million reads per sample, considering a 75 bp library fragment size. The sequencing pipeline, including library preparation and sample barcoding, was outsourced to the GeneCore Facility, EMBL, Heidelberg, Germany. Raw Illumina sequence reads obtained by the pair-end sequencing strategy were filtered and trimmed with Trimmomatic software.^30^ Filtered sequence reads were aligned with the human genome (genome build GRCh38) using the STAR aligner.^31^ The gene counts were indexed to the different families of gene transcripts by the BioMart data portal.^32^ Data were normalized using the variance-stabilizing transform in the DESeq2 package.^33^ Differential gene expression between working groups was determined by the Limma/Voom algorithm implemented in the iGEAK data processing platform for RNA-seq data.^34^

Small RNAs (miRs) were purified from the total RNA preparations and separated by capillary electrophoresis. Single-end sequencing libraries were prepared for Illumina sequencing using the recommendations from the manufacturer. Final data ensured an average number of 10 million reads per sample. The small RNA sequencing was also performed at the GeneCore Facility, EMBL, Heidelberg, Germany. miR detection and quantification were performed by the Chimira package^35^ using the miRBase 22.1 as a reference. Differential miR expression among sample groups was analysed by the DESeq2 and EdgeR algorithms, implemented in the NASQAR platform for high-throughput sequencing data analysis and visualization.^36^

### Endpoints

The main endpoint was to identity the miRs and related transcripts associated to the absence of reversal of LV hypertrophy to normal values according to indexed LV mass assigned to each sex, at 1-year follow-up. A secondary analysis considered as endpoint was a <14% reversion of mass from indexed values of baseline mass vs. ≥14%.^29^

Clinical events including all-cause death, heart failure, repeated hospitalizations, severe arrhythmia, stroke, and renal failure were examined at follow-up and their predictors at baseline.

### Statistical analysis

Categorical variables were presented as frequency rates/percentages and continuous variables as mean and SD. Categorical and continuous variables were compared using Pearson *χ*^2^ and *t*-tests, respectively. The comparison of means was performed using analysis of variance and correlation between means using Spearman’s test. Univariate analysis was assessed for all baseline clinical, biochemical, and imaging variables against the outcome of hypertrophy regression.

Working groups were defined according to late follow-up data, as exhibiting hypertrophy regression vs. absence of regression to normal values, as proposed as main outcomes. A secondary analysis was performed using, as outcomes, the regression of ≥ 14% or <14% from indexed mass values.^29^

Differential gene expression between working groups was determined as presented above. Criteria for the selection of significant differentially expressed genes included an adjusted *P* < 0.05 and log_2_(FC) < −0.6 or log_2_(FC) > 0.6. Myocardial tissue miR differential expression was assessed between working groups. miR targets were predicted with the mirDIP algorithm,^37^ considering a high-order probability for the occurrence of miR–mRNA interactions. Functional inverse correlations between downregulated mRNA transcripts and upregulated miRs were graphically depicted with the Navigator software.^38^

Multivariate logistic regression analysis was performed for the identification of independent predictors of hypertrophy non-regression. A *P* < 0.05 was considered significant. The statistical software used to analyse the overall data was SPSS version 26 (IBM).

### Ethics

The study followed the Declaration of Helsinki guidelines for biomedical research involving human subjects. The study protocol was approved by the Ethics Committee of the University Hospital, and all patients provided written informed consent.

## Results

### Hypertrophy regression after aortic valve replacement

**Figure 1:**
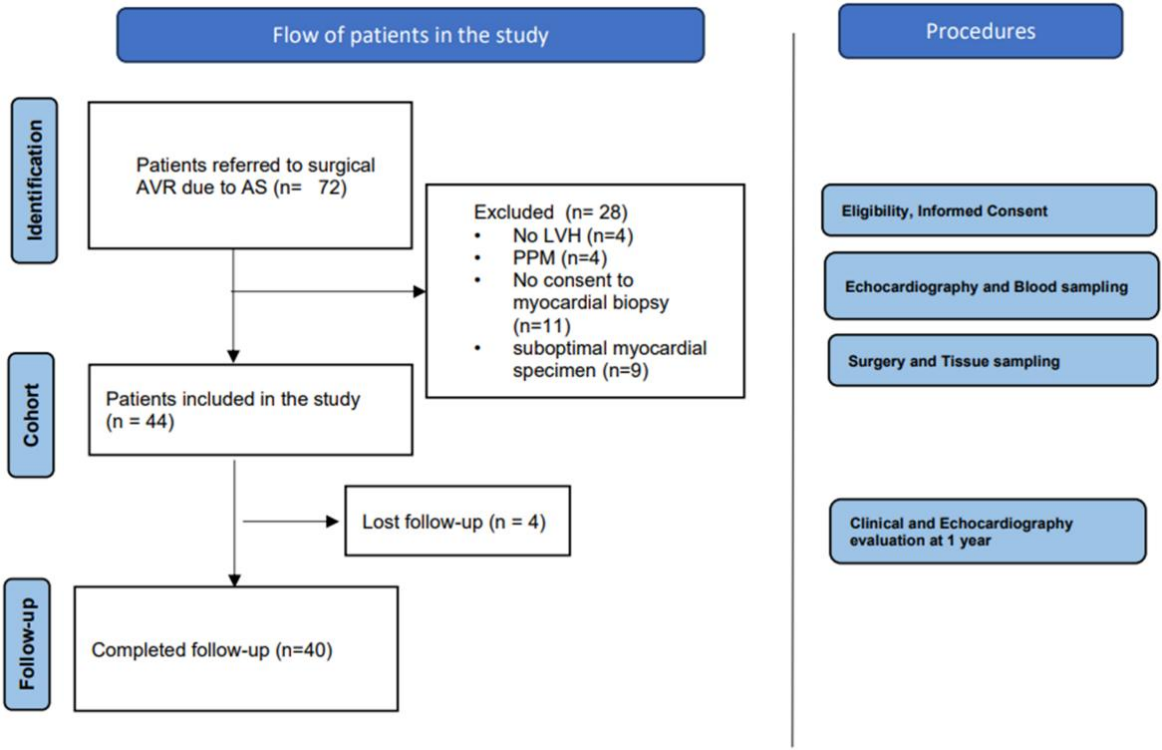
Flowchart of the included cohort. AVR, aortic valve replacement.

Among the 72 patients prospectively included in this study, 40 (52.5% women, 47.5% men, 74.7 ± 7 years old) were included for the purpose of miRs and transcriptomic analysis. As per protocol design, four patients were excluded due to the absence of baseline hypertrophy and additional four to the presence of patient–prosthesis mismatch on the early 1-month follow-up echocardiogram. From the remaining 64 patients, 9 patients were excluded due to inadequate tissue sample quality for analysis, 11 did not consent on myocardial biopsy, and 4 were lost for follow-up (*Figure 1*). Patients with hypertension were all controlled with appropriate therapy at baseline and at follow-up. Demographic, clinical, biochemical, and echocardiographic pre-operative data are presented in *Table 1*. Patients excluded from the study did not show significant differences from the included ones, regarding risk factors, severity of AS, and LV mass. All patients underwent stented aortic valve biological prosthesis implantation (Perceval, Intuity, Magna, and Trifecta).

**Table 1:** Clinical, echocardiographic, and biochemical variables in patients with iMass regression vs. no regression

Variables	*n* = 40	Reversal to normal^a^	No reversal to normal^a^	*P*	Reversal ≥ 14%^b^	No reversal to <14%^b^	*P*
		*n* = 19	*n* = 21		*n* = 17	*n* = 23	
Male sex, *n* (%)	19 (47.5)	10 (52.6%)	9 (42.9%)	0.54	10 (58.8%)	9 (39.1%)	0.22
Age, years	74 ± 7.3	75.11 ± 6.83	74.33 ± 7.85	0.74	74.24 ± 6.79	75.04 ± 7.79	0.73
BSA, m^2^	1.8 ± 0.2	1.78 ± 0.20	1.81 ± 0.23	0.67	1.82 ± 0.18	1.78 ± 0.23	0.5
Hypertension, *n* (%)	15 (37.5)	13 (68.4)	19 (90.5)	0.08	12 (70.6%)	20 (87.0%)	0.2
Diabetes, *n* (%)	13 (32.5)	6 (31.6)	7 (33.3)	0.91	6 (35.3%)	7 (30.4%)	0.75
Hyperlipidaemia, *n* (%)	30 (75)	15 (78.9)	15 (71.4)	0.58	15 (88.2%)	15 (65.2%)	0.1
AVA, cm^2^	0.97 ± 1.14	0.87 ± 0.12	1.16 ± 1.57	0.10	0.87 ± 0.14	1.12 ± 1.50	0.15
iAVA, cm^2^/m^2^	0.46 ± 0.08	0.44 ± 0.08	0.47 ± 0.08	0.34	0.44 ± 0.08	0.47 ± 0.09	0.22
Mass, g	269 ± 62	255.89 ± 59.25	281.24 ± 64.20	0.31	284.00 ± 53.99	258.26 ± 67.05	0.32
iMass, g/m^2^	151 ± 31	143.53 ± 28.00	157.71 ± 32.37	0.15	156.12 ± 26.69	147.17 ± 33.64	0.37
LVEDV, mL	102 ± 28.7	100.42 ± 29.72	104.38 ± 28.66	0.90	108.18 ± 31.16	98.30 ± 26.97	0.50
iLVEDV, mL/m^2^	57 ± 14	55.42 ± 14.47	59.14 ± 13.75	0.41	59.00 ± 15.62	56.17 ± 12.98	0.54
EF, %	59.5 ± 4.3	59.5 ± 3.1	59.4 ± 5.32	0.94	60.24 ± 4.18	58.91 ± 4.34	0.35
LAV, mL	79 ± 23	78.37 ± 24.91	79.10 ± 20.95	0.24	86.35 ± 25.95	73.13 ± 18.42	0.11
iLAV, mL/m^2^	44 ± 12	43.42 ± 12.09	44.19 ± 12.84	0.85	47.00 ± 12.34	41.48 ± 12.06	0.16
SV, mL	66 ± 24	615.95 ± 290.16	715.38 ± 183.94	0.06	671.71 ± 240.52	652.30 ± 251.89	0.82
iSV, mL/m^2^	41 ± 2.8	41.89 ± 2.49	40.90 ± 3.15	0.18	41.35 ± 2.52	41.39 ± 3.14	0.21
GLS, %	14.2 ± 3.3	14.23 ± 3.36	14.22 ± 3.38	0.99	13.94 ± 2.69	14.44 ± 3.78	0.64
GWE, %	84 ± 4.4	84.8 ± 5.44	83.76 ± 3.25	0.44	84.0 ± 5.46	84.48 ± 3.54	0.74
cTNT-hs, ng/L	18.5 ± 7.9	12.74 ± 3.57	23.71 ± 7.15	<0.001	14.24 ± 4.94	21.65 ± 8.33	0.002
NT-proBNP, pg/mL	1533 ± 202	1302 ± 886	1765 ± 2844	0.72	1229 ± 849	1959 ± 3128	0.56
Haemoglobin, g/dL	13 ± 1.6	12.77 ± 1.42	13.24 ± 1.79	0.37	12.79 ± 1.37	13.18 ± 1.79	0.45
GFR, mL/min/1.73 m^2^	74 18.6	77.21 ± 16.81	71.38 ± 20.10	0.33	80.88 ± 14.04	69.17 ± 20.25	0.048

BSA, body surface area; AVA, aortic valve area; iAVA, indexed body area to BSA; Mass, LV mass; iMass, indexed LV mass to BSA; LVEDV, left ventricular end-diastolic volume; iLVEDV, indexed LVSDV to BSA; EF, ejection fraction; LAV, left atrial volume; iLVA, indexed LVA to BSA; GLS, global longitudinal strain; GWE, global myocardial work efficiency; cTNT-hs, high-sensitivity troponin T; GFR, glomerular filtration rate.

^a^Reversal/no reversal to normal iMass according to sex.

^b^Reversal/no reversal of iMass to ≥14% vs. <14%.

At a mean follow-up of 11 ± 4 months after surgical AVR, a significant reduction in the overall LV mass (263 ± 59 vs. 206 ± 58 g, *P* < 0.0001) and in the iLVM (147 ± 31 vs. 113 ± 32 g/m^2^, *P* = 0.001) was observed for all the population with a mean reduction of 15 ± 11%. However, in 52.5%, there was an absence of reverse remodelling in hypertrophy to normal indexed mass values, according to sex. Moreover, in 57.5%, there was <14% in indexed mass regression. During follow-up, no differences were found regarding the LV EF (59.4 ± 4 vs. 62 ± 6%, *P* = 0.2). All included patients showed the absence of aortic prosthesis dysfunction with a mean trans-prosthetic gradient of 18 ± 5 mmHg.

### Clinical endpoints

There was no mortality at follow-up. Two patients were hospitalized for heart failure at 7 and 9 months after surgery, both with hypertrophy and preserved EF. Eight patients underwent a pacemaker insertion due to advanced atrioventricular heart block during surgery or at the early post-operative period.

### Clinical, biochemical, and echocardiographic variables in hypertrophy according to myocardial regression

Univariate analysis (*Table 1*) showed no difference between baseline clinical or echocardiographic variables in patients presenting the absence of LV mass regression to normal vs. the ones with regression. The same was found for patients with <14% regression in the iMass values (*Table 1*). Considering the biochemical data, cTNT-hs was significantly higher in the group with no reversal in comparison with the one with reverse remodelling, with the same holding true for the patients with <14% reversal vs. with ≥14%. GFR was lower in patients with <14% reversal vs. with the ones with ≥14% iMass reversal. All remaining variables were not different between the two groups.

### Transcriptomic analysis

**Figure 2:**
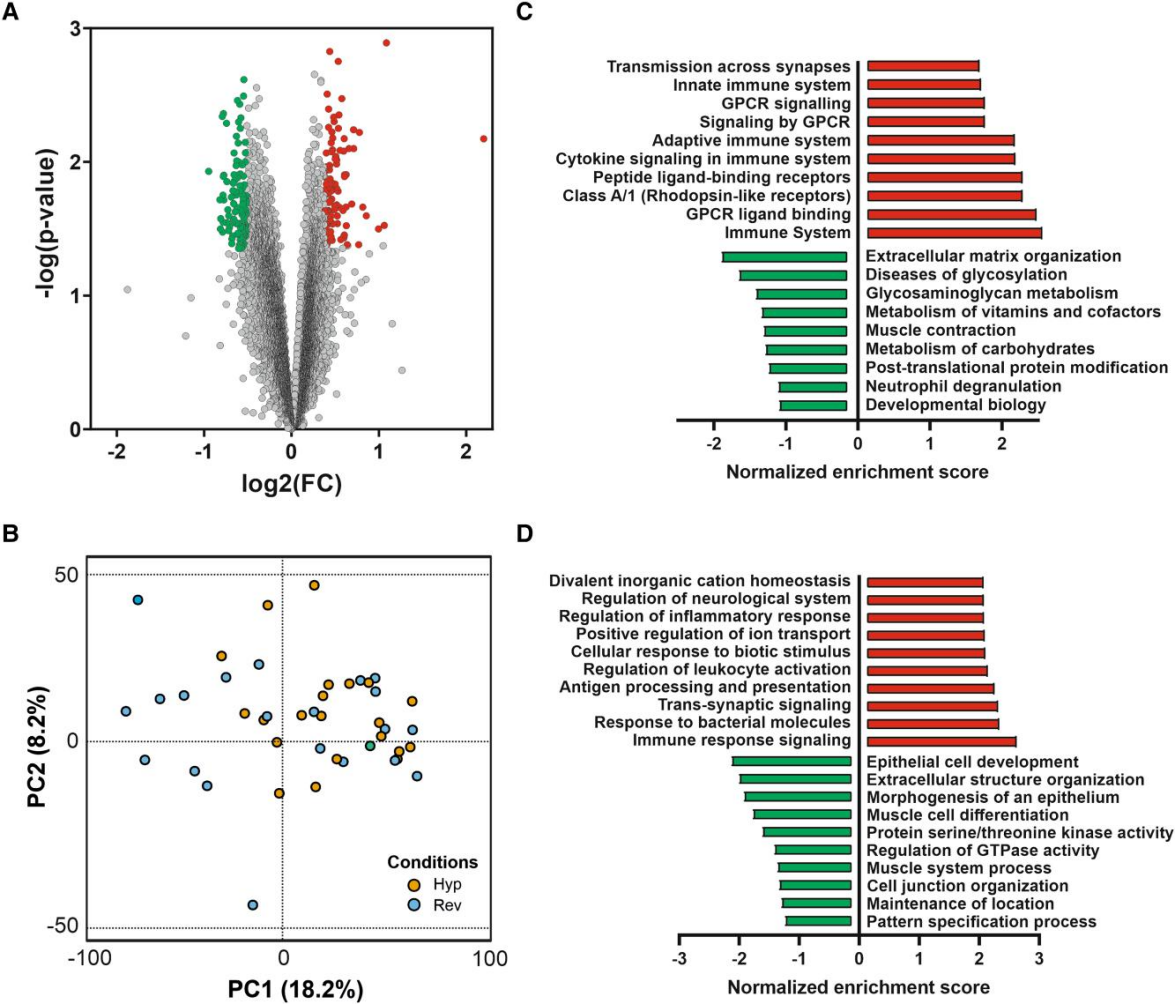
Differential expression and functional analysis of coding mRNAs in the patients with reverse myocardial remodelling after aortic valve replacement, when compared with the persistent phenotype. (*A*) Volcano plot of the differentially expressed mRNAs. (*B*) Principal component analysis (PCA) analysis (Rev: patients with reverse myocardial remodelling after aortic valve replacement; Hyp: patients with persistent hypertrophic phenotype after aortic valve replacement). (*C*) Gene set enrichment analysis of the biochemical pathways integrated within the Reactome database, performed by WebGestalt application.^39^ (*D*) Gene set enrichment analysis of the gene ontology terms selected by biological processes, performed by WebGestalt application.

We used transcriptomic analysis of the myocardial samples obtained before AVR to determine the differentially expressed mRNA transcripts in patients with hypertrophy reversion compared with the group of sustained phenotype observed in the follow-up. This transcriptomic pattern is mainly characterized by downregulation of gene expression (67 downregulated gene transcripts vs. 36 upregulated ones) (*Figure 2A*). Despite the control and exhaustive characterization of clinical variables in the definition of the analysed groups of patients, the gene expression pattern did not provide a clear group stratification when analysed by principal component analysis (*Figure 2B*).

Interestingly, when we performed a gene set enrichment analysis of the differentially expressed transcripts, we found a negative enrichment score in pathways and biological processed related to cell proliferation, metabolism of carbohydrates, muscle contraction, and extracellular matrix organization (*Figure 2C* and *D*). Positively enriched processes included immune system response, cytokine signalling, ion transport, leucocyte activation, and regulation of inflammatory responses (*Figure 2C* and *D*).

### miR expression and functional analysis

**Figure 3:**
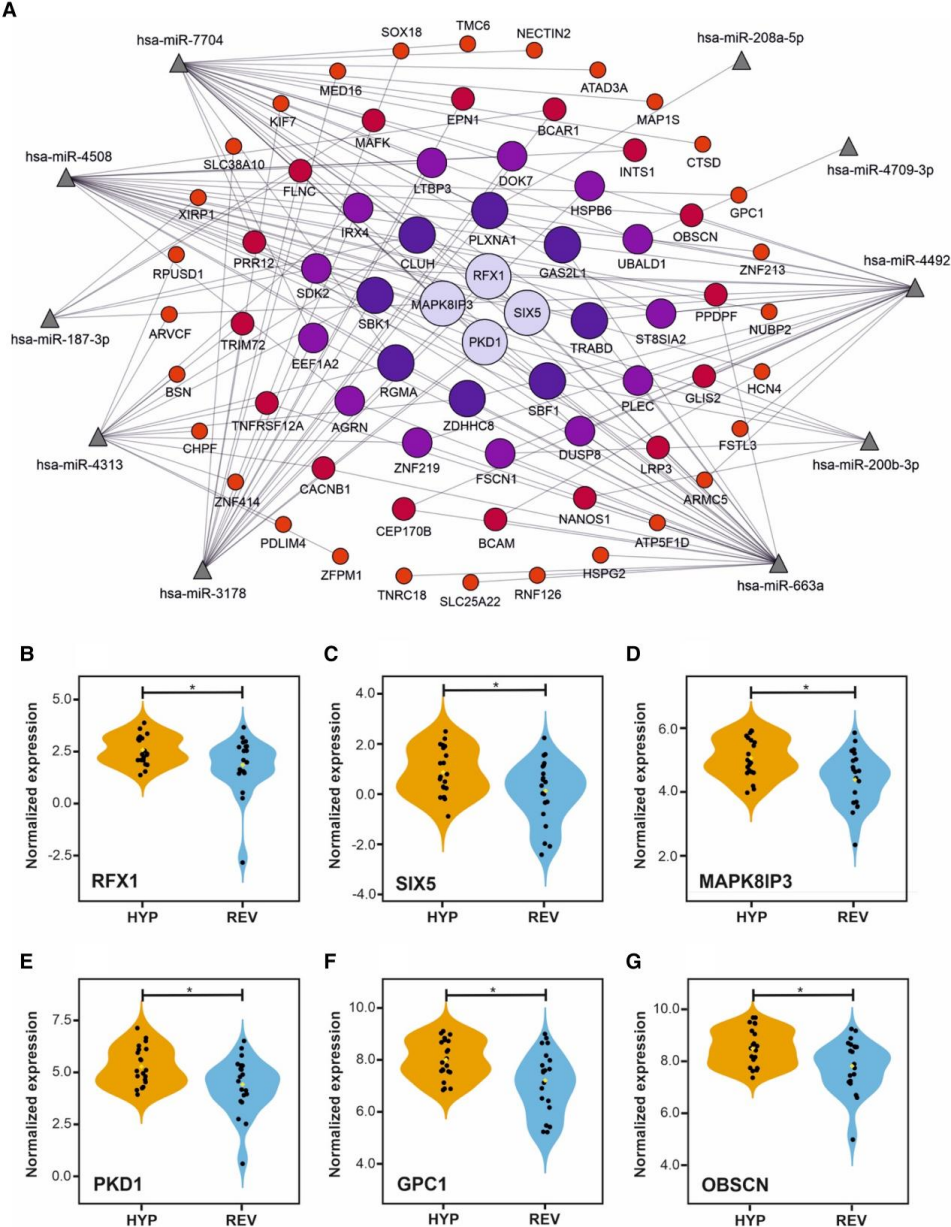
Functional analysis of the transcriptomic data from the selected patients. (*A*) mRNA–miR interaction networks established in patients with reverse myocardial remodelling and no detectable hypertrophy. Upregulated miRs, represented by triangles, are connected with lines to their cognate targets as predicted by the mirDIP algorithm. Depicted gene targets, represented by circles, are downregulated in the remodelled myocardium, suggesting a possible functional connection between them. The size of the gene circle symbols is proportional to the number of miRs targeting a particular mRNA. (*B–E*) The normalized gene expression values in both groups of patients of the gene transcripts predicted to be regulated by five upregulated miRNAs. (*F* and *G*) The normalized gene expression of gene transcripts targeted by miR-4709-3p. HYP, patients with hypertrophy after follow-up; REV, patients with reverse remodelling after follow-up. *P < 0.05.

We detected around 300 miRs by small RNA-seq in the myocardial biopsy samples, including 29 that were differentially expressed in the patients with reversed hypertrophy in comparison to the ones with persistent hypertrophy (*Table 2*). Putative human targets for the differentially expressed miRs were predicted by using mirDIP algorithm. To gain insights about the possible functional roles of the differentially expressed miRs in patients with reverse myocardial remodelling, we built a miR-centred regulatory network with the upregulated miRs and their downregulated cognate targets as predicted by mirDIP (*Figure 3*). Considering the upregulated miRs as a transcriptional signal, those targets that are potentially regulated by more than one miR should have an increased relevance in the context of the studied system. Following this trend, we determined that the downregulated gene transcripts RFX1, SIX5, MAPK8IP3, and PKD1 are simultaneously regulated by five of the upregulated miRs, suggesting their intrinsic importance in the process of hypertrophy reversion.

**Table 2:**
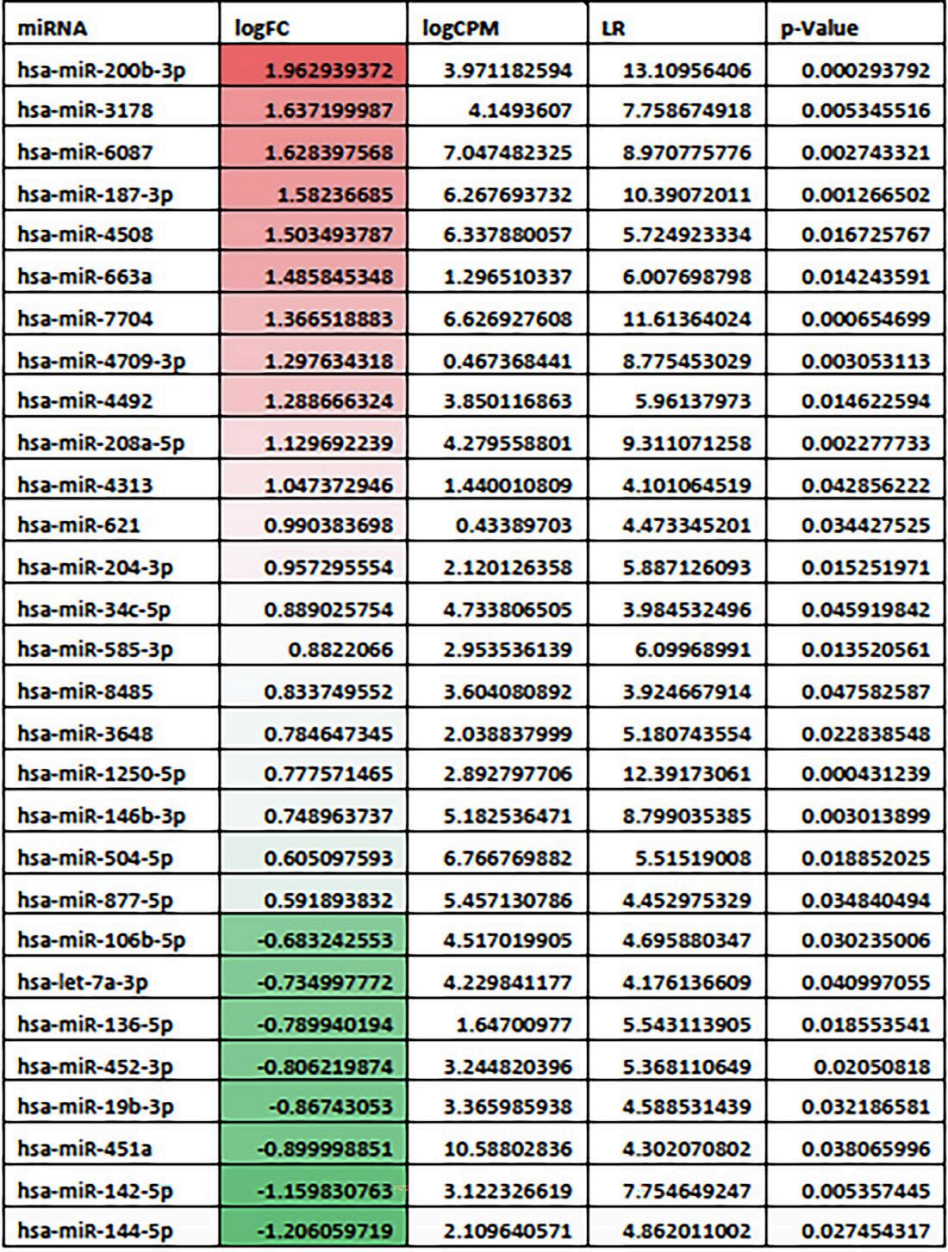
Differentially expressed miRNAs in comparison between the myocardium from patients with reversed hypertrophic phenotype vs. patients with persistent hypertrophy determined by the EdgeR algorithm

Upregulated miRNAs are represented in red and downregulated ones are highlighted in green.

logFC, log_2_ fold change between the groups; logCPM, the average log_2_ counts per million; LR, likelihood ratio statistics; *P*-value, the two-sided *P*-value.

### Correlation of mass regression with clinical, biochemical, and miR expression

Considering the two groups, with and without LV mass regression,^2^ by univariate analysis, only hsa-miR-4709-3p was significantly differentially expressed in the group without mass regression vs. the one with regression (1.00 ± 1.22 vs. 2.47 ± 1.78, *P* = 0.005). Considering the group classified with <14% regression vs. the one with ≥14% regression from mass values, has-miR-4709-3p was also significantly expressed (1.09 ± 1.31 vs. 2.53 ± 1.77, *P* = 0.005) (*Table 3*).

**Table 3:** Comparison of miRNAs in patients with reversal hypertrophy vs. no reversal

miRNA	Reversal to normal^a^	No reversal to normal^a^	*P*	Reversal ≥ 14%^b^	No reversal to <14%^b^	*P*
	*n* = 19	*n* = 21		*n* = 17	*n* = 23	
has-miR-200b-3p	51.42 ± 160.75	13.14 ± 5.28	0.28	54.94 ± 170.15	13.87 ± 5.11	0.25
has-miR-3178	61.21 ± 199.10	22.52 ± 34.47	0.39	67.18 ± 210.32	21.48 ± 33.05	0.31
has-miR-6087	276.37 ± 590.68	322.62 ± 732.66	0.83	325.24 ± 620.20	282.48 ± 702.76	0.84
has-miR-187-3p	267.63 ± 766.20	111.90 ± 82.36	0.36	294.59 ± 808.31	105.52 ± 80.17	0.27
has-miR-4508	203.21 ± 582.71	178.90 ± 396.43	0.88	234.35 ± 612.92	158.00 ± 380.98	0.63
has-miR-663a	5.79 ± 13.58	2.85 ± 2.49	0.34	6.47 ± 14.29	2.60 ± 2.31	0.21
has-miR-7704	303.16 ± 683.75	167.57 ± 131.85	0.38	331.82 ± 720.09	158.17 ± 127.02	0.26
has-miR-4709-3p	2.47 ± 1.78	1.00 ± 1.22	0.005	2.53 ± 1.77	1.09 ± 1.31	0.005
has-miR-4492	30.42 ± 48.77	36.86 ± 81.30	0.77	36.12 ± 50.42	32.09 ± 78.21	0.85
has-miR-208a-5p	61.63 ± 143.80	33.62 ± 14.04	0.38	65.65 ± 151.78	33.09 ± 15.11	0.31
has-miR-4313	6.05 ± 10.49	3.62 ± 4.59	0.36	6.76 ± 10.95	3.30 ± 4.39	0.18
has-miR-621	1.68 ± 1.38	1.57 ± 2.13	0.84	1.94 ± 1.92	1.39 ± 1.70	0.34
has-miR-204-3p	8.16 ± 6.77	8.43 ± 8.93	0.92	8.29 ± 7.10	8.30 ± 8.56	1
has-miR-34c-5p	82.37 ± 156.39	38.57 ± 22.20	0.21	83.53 ± 166.31	41.52 ± 20.72	0.24
has-miR-585-3p	20.84 ± 24.79	14.14 ± 7.91	0.25	22.65 ± 25.31	13.39 ± 8.72	0.11
has-miR-8485	22.00 ± 36.94	30.38 ± 39.60	0.49	24.65 ± 38.97	27.70 ± 38.28	0.81
has-miR-3648	8.74 ± 6.67	8.48 ± 9.46	0.92	8.71 ± 7.05	8.52 ± 9.03	0.94
has-miR-1250-5p	18.42 ± 10.50	14.52 ± 6.84	0.17	17.94 ± 11.08	15.22 ± 6.86	0.34
has-miR-146b-3p	85.95 ± 65.29	80.71 ± 42.71	0.76	89.12 ± 68.11	78.83 ± 41.68	0.56
has-miR-504-5p	204.00 ± 79.59	295.71 ± 206.62	0.08	229.71 ± 95.98	268.74 ± 201.07	0.46
has-miR-877-5p	95.79 ± 66.89	103.67 ± 63.45	0.7	100.59 ± 68.83	99.43 ± 62.47	0.96
has-miR-106b-5p	43.00 ± 27.56	67.71 ± 58.87	0.1	43.24 ± 29.49	65.39 ± 56.55	0.15
has-let7a-3p	43.74 ± 29.73	47.43 ± 48.38	0.78	40.82 ± 26.58	49.26 ± 48.08	0.52
has-miR-136-5p	4.84 ± 3.99	6.48 ± 5.24	0.28	4.47 ± 4.26	6.61 ± 4.90	0.16
has-miR-452-3p	17.00 ± 8.24	26.52 ± 27.48	0.15	16.59 ± 8.74	26.00 ± 26.23	0.16
has-miR-19b-3p	20.21 ± 16.76	27.67 ± 36.49	0.42	21.47 ± 17.33	26.09 ± 35.18	0.62
has-miR-451a	2955.42 ± 2285.15	4898.86 ± 6132.96	0.2	2547.94 ± 2021.91	5031.04 ± 5870.78	0.1
has-miR-142-5p	15.11 ± 9.45	25.48 ± 35.04	0.22	15.94 ± 10.26	23.96 ± 33.66	0.35
has-miR-144-5p	6.00 ± 5.56	11.38 ± 20.17	0.27	5.06 ± 5.08	11.61 ± 19.24	0.18

^a^Reversal/no reversal to normal iMass according to sex.

^b^Reversal/no reversal of iMass to ≥14% vs. <14%.

A logistic regression was performed to ascertain the effects of pre-operative variables on the likelihood that participants have no regression or <14% mass regression. This analysis showed that has-miR-4709-3p and cTNT-hs were independently associated with hypertrophy regression among all variables and using both outcomes (*Table 4*). has-miR-4709-3p was significant in one of the logistic regression models, while troponin was significant in both models. Therefore, troponin is an independent predictor of no hypertrophy regression independent of miR or basal indexed LV mass (marker of the severity of basal LV hypertrophy). On the other hand, miR-4709-3p predicted the absence of full reversal independently of troponin and basal indexed LV mass. The indexed ventricular mass at baseline was also an independent positive predictor for <14% regression. The model explained 82.7 and 69.0% (Nagelkerke *R*^2^) of the variance of mass regression and of ≥14% mass regression, respectively, and correctly classified 89.5 and 76.5% of cases.

**Table 4:** Predictors by logistic regression of no hypertrophy regression and <14% regression at 1-year follow-up in patients undergoing aortic valve surgery for aortic stenosis

	Model 1: no reversal			Model 2: reversal < 14%		
Variable	Coefficient *B*	Wald	*P*	Coefficient *B*	Wald	*P*
hs-TNT	−0.579	8.467	0.04	−0.495	7.543	0.006
miR-4709-3p	0.958	4.923	0.26	1.076	6.343	0.012
Bas iMass	—	—	—	0.075	6.566	−0.10

hs-TNT, high-sensitivity troponin T; Bas iMass, baseline pre-operative left ventricular mass indexed to body surface area.

From all considered variables, hsa-miR-4709-3p (*R* = 0.34, *P* = 0.026) and cTNT-hs (*R* = −0.40, *P* = 0.009) were moderately correlated with the percentage of hypertrophy regression observed in the patients.

## Discussion

In our prospective study of patients with severe AS undergoing successful and aortic valve surgery, about 50% showed the absence of LV hypertrophy regression at 1-year follow-up. We assessed predictors of mass regression, considering a list of clinical echocardiographic and biochemical variables. Using cardiac tissues, we were also able to characterize a fingerprint composed by 29 differentially expressed miRNAs.

Our findings determined that miR-4709-3p was a negative independent predictor together with cTNT-hs that was a positive independent predictor of the absence of hypertrophy regression after surgical AS relieve. These findings were consistent to both outcomes considered in our study, namely, the absence of regression to normal LV mass values, and the regression of <14% of indexed mass, a cut-off with prognostic significance, as proposed by a recent study.^29^ We used these two different outcomes, since results could bring new perspectives for future works. Nevertheless, the independent predictors were the same in both outcomes.

Post-operative left ventricular hypertrophy has been previously associated with major adverse cardiac events.^10,12,13^ In patients with the absence of both patient–prosthesis mismatch and prosthesis dysfunction, mechanisms underlying hypertrophy non-reversal are poorly characterized, ranging from sex, haemodynamic, and genetics, but findings are inconsistent.^8^ Identifying predictors of non-reversal could open the opportunity for acting early over those factors and improve prognosis. In our study, baseline demographic variables, risk factors, and echocardiographic data, including ventricular volumes and mass, EF, myocardial strain, and myocardial work, were not predictors of mass regression. Concomitant hypertension was excluded as main cause of non-reversal, since blood pressure was found with normal values along follow-up. The stentless biological aortic prosthesis remained with normal function as per study design, thus also being excluded as causing LV hypertrophy persistence.

Considering the previous data, we decided to assess whether miRs could act as potential modulators of the hypertrophic phenotype reversion. Considering the observed downregulated pattern of gene expression in the myocardium of patients that showed a reverted hypertrophic phenotype after intervention, we thus hypothesized about the role of tissue miRs in the establishment of this gene expression fingerprint. We were able to detect 29 differentially expressed miRs, being 21 upregulated and 8 downregulated in the patients showing reversion of the hypertrophic phenotype.

Among the upregulated miRs determined in our study, miR-200b has been described as a profibrotic agent that is controlled by the TRF-beta axis and characterized as downregulated in hypertrophic scars.^40^ miR-6087 also seems to be a post-transcriptional regulator of cardiomyogenic differentiation.^41^ On the other hand, downregulated miR144-3p and 451 were differentially expressed in patients with hypertrophy phenotype. The miR144/451 family was associated in previous studies with extracellular matrix remodelling and negative regulation of hypertrophy and autophagy.^42^ miR19b-3p, a downregulated miR detected in patients with reverse hypertrophic phenotype, has been previously characterized in rat models as a pro-hypertrophic miR, by observing the induction of hypertrophy in rat neonatal cardiomyocytes overexpressing miR-19a/b.^43^ In this model, miR-19a/b exerted its regulatory action by targeting Atrogin1 and Murf1, modulating the signalling pathways calcineurin/NFAT and PCK, respectively. In a human study,^22^ upregulated miR133 was a moderate predictor of hypertrophy regression in operated AS patients. Results are not directly comparable to ours, since we assessed the most representative miR from a setting of 300 detected miRs in the myocardium.

We determined that the upregulated miR-4709-3p is an independent predictor of hypertrophy reversal. There are still few studies regarding this miR. In a human model of hypertensive women, miR-4709-3p was found to repress the expression of RAC1 mRNA transcript.^44^ RAC1, a central small GTP-binding protein, regulates cell proliferation, survival, migration, and trafficking and triggers NADPH oxidase, which contributes to oxidative stress. Over-expression of RAC1 was found to be an essential component of the signalling pathway in the myocardium initiating LV hypertrophy.^45,46^ We hypothesize that this pathway could be implicated in the hypertrophy reversal observed in our study, although definite conclusions need to be confirmed in further studies.

Furthermore, considering the upregulated miRs as a transcriptional signal, we found that the downregulated gene transcripts RFX1, SIX5, MAPK8IF3, and PKD1 are simultaneously regulated by five of the upregulated miRs, suggesting their intrinsic importance in the process of hypertrophy reversion. MAPK8/9 signalling has already been described as an important inducer of pathological hypertrophy, as determined by using cellular and animal models.^47^ However, the most interesting gene is PKD1. By using animal models, Bossuyt *et al.*^48^ determined that the upregulation of PKD1 is involved in the hypertrophic response and in the potassium channel downregulation observed in heart failure. Interestingly, PKD1 also regulates insulin and glycaemia,^49^ which is in line with our transcriptomic findings. Moreover, the reduction of miR-4709-3p in persistent LV hypertrophy patients occurs with consistently increased level of transcripts GPC1 and OBSC, which are both regulated by miR-4709-3p, and encode glypican-1 and obscurin, respectively. Glypican-1 has been associated with cardiac voltage-activated K^+^ currents and LV hypertrophy,^50^ while obscurin associates with LV hypertrophy and hypertrophic cardiomyopathy.^51^

The functional analysis of the transcriptomic data allowed us to find a negative enrichment score in pathways and biological processes related to cell proliferation, muscle contraction, extracellular matrix organization, and metabolism of carbohydrates. Interestingly, previous evidence showed that during the development of hypertrophic cardiomyopathy, the heart returns to foetal energy metabolism where cells utilize more glucose instead of fatty acids as a source of energy.^52^ Metabolism of glucose can increase synthesis of the extracellular glycosaminoglycan hyaluronan, which has been shown to be involved in the development of cardiac hypertrophy and fibrosis.^53,54^ Hyperglycaemia and diabetes were found to relate with ventricular hypertrophy and dysfunction through mechanisms not well clarified.^55^ We hypothesize that these biological processes involving the carbohydrate metabolism could occur as a consequence of the hypertrophy development and not necessarily as an underlying causal mechanism.

Finally, in our study, cTNT-hs was a predictor of persistent hypertrophy. High-sensitivity troponins (cTNT-hs) were previously found to relate to LV hypertrophic response, proposed as markers of LV decompensation, myocardial fibrosis, and adverse events in patients with AS.^56^ High-sensitivity troponin T levels were however modestly increased, and its usefulness as biomarkers in a clinical setting awaits further assessment in a larger population.

The main limitation of this study is the size of the population. However, we consider that findings are robust taking into account not only the homogeneity of the cohort but also that a myocardial tissue sample was used for miR quantification, allowing to extract the ones differentially expressed according to the outcomes. Second, a longer follow-up should be undertaken in order to understand the role of miRs on the long-term evolution of hypertrophy and the associated clinical major adverse events.

## Conclusions

In this study, we hypothesized about the role of myocardial microRNAs in the prediction of hypertrophy reversal after AS release. We found that hsa-miR-4709-3p is an independent predictor of this reversion phenotype, downregulating gene transcripts GPC1 and OBSC, which encode glypican-1 and obscurin, respectively, suggested to relate to hypertrophy development. Furthermore, establishing an RNA regulatory network, we determined that gene transcripts RFX1, SIX5, MAPK8IF3, and PKD1 were predicted as simultaneous targets of five upregulated miRs, suggesting their importance in the hypertrophy remodelling. The transcriptomic analysis of the myocardial samples yielded mRNA transcripts related to the metabolism of carbohydrates, indirectly related hypertrophy. The ultimate hypertrophy reversal process will likely depend from a complex network where miRNAs may have an important role, allowing a potential opportunity for therapy, namely, using chemically synthetized miR mimics and other interventional strategies.

## Lead author biography


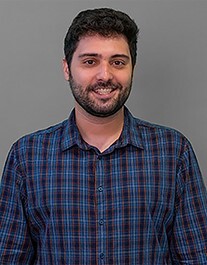


André F. Gabriel holds a Bachelor’s in Genetics and Biotechnology and a Master’s in Molecular and Cell Biology. Currently, André is a researcher at the Institute for Molecular Medicine and a PhD candidate in Biomedical Sciences at Lisbon’s Faculty of Medicine. His academic journey, fuelled by an unyielding passion for understanding the intricacies of biological systems, has culminated in a focused pursuit of the regulatory networks of non-coding RNAs, seeking to decode their roles in disease progression. His research delves deep into the dynamics of circular RNAs and RNA-binding protein interactions, offering profound insights into the molecular mechanisms shaping disease pathogenesis.

## Data Availability

Next-generation sequencing data were deposited in the Short Read Archive (SRA) database under the BioProject reference PRJNA1098010. The data underlying this article will be shared upon reasonable request to the corresponding author.
